# Addressing the needs of Ethiopia’s street homeless women of reproductive age in the health and social protection policy: a qualitative study

**DOI:** 10.1186/s12939-023-01874-x

**Published:** 2023-05-04

**Authors:** Kalkidan Yohannes, Mats Målqvist, Hannah Bradby, Yemane Berhane, Sibylle Herzig van Wees

**Affiliations:** 1 SWEDESD- Department of Women’s and Children’s Health, Uppsala University, Uppsala, Sweden; 2 WOMHER- Women’s Mental Health During the Reproductive Lifespan, Interdisciplinary Research Center, Uppsala University, Uppsala, Sweden; 3 Department of Psychiatry, College of Health and Medical Science, Dilla University, Dilla, Ethiopia; 4 Department of Sociology, Uppsala University, Uppsala, Sweden; 5 Addis Continental Institute of Public Health, Addis Ababa, Ethiopia; 6 Department of Global Public Health, Karolinska Institute, Stockholm, Sweden

**Keywords:** Health, Well-being, Street homeless women, Street homelessness, Ethiopia, Qualitative research, Policy agenda, Shiffman and Smith’s framework, Political priority, Low and Middle-Income Countries

## Abstract

**Introduction:**

Globally, homelessness is a growing concern, and homeless women of reproductive age are particularly vulnerable to adverse physical, mental, and reproductive health conditions, including violence. Although Ethiopia has many homeless individuals, the topic has received little attention in the policy arena. Therefore, we aimed to understand the reason for the lack of attention, with particular emphasis on women of reproductive age.

**Methods:**

This is a qualitative study; 34 participants from governmental and non-governmental organisations responsible for addressing homeless individuals’ needs participated in in-depth interviews. A deductive analysis of the interview materials was applied using Shiffman and Smith’s political prioritisation framework.

**Results:**

Several factors contributed to the underrepresentation of homeless women’s health and well-being needs in the policy context. Although many governmental and non-governmental organisations contributed to the homeless-focused programme, there was little collaboration and no unifying leadership. Moreover, there was insufficient advocacy and mobilisation to pressure national leaders. Concerning ideas, there was no consensus regarding the definition of and solution to homeless women’s health and social protection issues. Regarding political contexts and issue characteristics, a lack of a well-established structure, a paucity of information on the number of homeless women and the severity of their health situations relative to other problems, and the lack of clear indicators prevented this issue from gaining political priority.

**Conclusions:**

To prioritise the health and well-being of homeless women, the government should form a unifying collaboration and a governance structure that addresses the unmet needs of these women. It is imperative to divide responsibilities and explicitly include homeless people and services targeted for them in the national health and social protection implementation documents. Further, generating consensus on framing the problems and solutions and establishing indicators for assessing the situation is vital.

## Introduction

Homelessness has been recognised as a global problem that affects both high and low-income countries [1, 2]. Globally, the United Nations estimates that 1.6 billion people lack adequate shelter, and more than 100 million live without a roof over their heads [3]. In Ethiopia, a country with more than 117 million inhabitants, homelessness is widespread in major cities [4–6].

Homelessness is characterised by a lack of adequate housing [7]. Individuals experiencing homelessness lack safe and secure housing and often live in shelters, streets, or other public spaces [7, 8]. Despite this, no single, globally agreed definition of homelessness exists in either policy or research [9]. For example, it is defined differently in each country [9, 10].

Homeless people can be classified into four categories based on their living conditions: roofless (street homeless), houseless, inadequate, and insecure [8]. Those living on the streets without shelter or staying in a night shelter are considered street homeless [8]. In contrast, the absence of a permanent residence and the frequent need to move house characterise those who are houseless [8]. Those who reside in a private dwelling but do not report a permanent address in their census form fall into this category [8, 10]. People are considered to live in insecure or inadequate housing if eviction, experience domestic violence or extreme overcrowding threatens them, or if the dwelling is unfit for a purpose [8, 10]. Street homeless women are the focus of this study. This group has no access to permanent housing and lives on the streets alone or with their families [8].

Homelessness is a complex global public health issue and a significant driver of poor health [11, 12]. It results from accumulating adverse social and economic conditions [13]. Health inequalities are associated with homelessness [14]; homeless people have a shorter life expectancy [14–16], a higher frequency of health problems [16–18], and a higher frequency of use of emergency hospital services than the general population [19]. Hence, homelessness may serve as both a cause and a consequence of poor health [18, 20]; the state of being homeless can affect the homeless person’s physical, mental, and social well-being [15, 20–22]. In addition, a high prevalence of substance use disorders, severe mental disorders, and suicide is reported among homeless women [21, 23, 24].

In addition to struggling with a lack of basic needs and mental health problems [25, 26], homeless women in Ethiopia face limited access to reproductive health services [27]. This lack of access to services exposes women to sexually transmitted infections, unprotected sex, unwanted pregnancy, and unsafe abortion [28]. Furthermore, injuries occasioned by homelessness are gendered, with homeless women suffering differently from homeless men [29].

Despite this, international and national leaders place varying degrees of importance on health issues [30]. A significant challenge facing health policy decision-makers worldwide is setting priorities for health interventions [31]. Developing a political priority involves deciding how healthcare resources should be allocated among competing programmes or individuals [32]. This process is inherently value-laden and political [33], requiring credible evidence, responsible institutions, and fair procedures [34].

Political priority is often a prerequisite for effectively addressing neglected public health problems, as evidenced by policy attention and resources invested in the issue [30, 35]. Setting priorities is vital in health policy design and crucial in low-income countries with limited resources [36]. Moreover, these countries may face further obstacles to priority setting, such as political instability, insufficiently developed social sectors, weak institutions, and significant social inequalities [37].

## Conceptual framework

In this study, Shiffman and Smith’s framework was used to explore the policy context that determines the political prioritisation of health and well-being for street homeless women [30]. The framework focuses on the process of setting global health priorities. Still, it has proved helpful in explaining the political process of prioritisation in different international, national, and subnational settings [38]. In particular, this framework has been used to study priority-setting processes in low-resource settings throughout Asia, Latin America, and sub-Saharan Africa, demonstrating its usefulness in studying health policy in resource-limited settings [39, 40].

Shiffman and Smith describe four factors that influence political prioritisation [30]. These are (1) actor power, (2) ideas, (3) political contexts, and (4) issue characteristics [30]. Actor power refers to the influence of the groups and individuals involved in a particular issue. The factors considered are the degree of cohesion among individual networks, the effectiveness of organisations or co-ordination mechanisms, and the number of individuals capable of uniting the policy community [30]. Ideas refer to how those involved understand and portray the issue [30]. Political context refers to the environment in which actors operate. Furthermore, issue characteristics in this framework refer to the features of the problem [30].

## Health, social protection, and gender in the Ethiopian policy context

A link exists between homelessness and women in three policy areas: health [41], social protection [42], and gender [43, 44]. A variety of Ethiopian policies, plans, and strategies address these three issues. Several national health implementation guidelines and strategies address issues pertinent to women’s health and gender [44–50].

In national health policy, realistic goals and means to achieve them are proposed based on the premise that sound health is a prerequisite for enjoying life and work [41]. However, there is no indication as to whether the homeless are included. Aside from the national mental health strategy [48] and the family planning guidelines [45], no specific intervention is outlined for homeless individuals in these health-related documents. The mental health strategy mentions interventions such as rehabilitation for street homeless individuals [48]. Further, the family planning guideline [45] recommends innovative family planning solutions. However, no definition of an innovative approach or further details is provided in the document.

Ethiopia has approved a document to eliminate Human Immunodeficiency Virus (HIV), hepatitis B, and syphilis transmission from mother to child [46]. In this document, services are targeted at women and girls, disabled individuals, refugees, HIV-positive individuals, and minorities [46]. However, street homeless individuals are not explicitly mentioned [46]. The country also has menstrual hygiene management [48] and antenatal care guidelines [47]. Nevertheless, these documents do not specifically address the issue of homeless women.

As part of its policy objectives, the government of Ethiopia has prioritised social protection, which has been evaluated in various academic and policy contexts [51, 52]. Through a gender lens and life cycle approach, it thoroughly reviews Ethiopia’s poverty and vulnerability context [51].

Social protection policy and strategy identify three main groups of poor and vulnerable individuals [42, 51]. The first group consists of individuals who live below the poverty line. The second group comprises families that experience poverty on more than one level, finding it challenging, for example, to obtain health, nutrition, water, sanitation, and education essentials. The third group contains those so poor that they are excluded from social and community activities due to their financial and/or physical or mental health situation [51]. Poor people may be deprived or at risk of vulnerability in these dimensions [42, 51].

Having a social protection strategy in place has proven to effectively reduce poverty and vulnerability [51]. In line with this, Ethiopia’s National Social Protection Policy and Strategy does contain a definition of vulnerability [42, 51]. However, neither the definition nor the target population list mentions homeless individuals [42, 51].

The Ministry of Health has developed guidelines for gender mainstreaming in all health sector programmes [44]. Besides this, the national documents listed above emphasise equity consciousness and gender awareness. However, only two documents out of nine identify homeless women as a target population and mention their needs explicitly [45, 48].

In summary, several terms repeatedly appear throughout the above documents, including ‘vulnerable’, ‘urban poor’, and ‘people with special needs’. Because vulnerability is a complex and contested concept [53], it may be challenging to determine which definition applies to homeless individuals, particularly since these documents do not explicitly mention them.

Therefore, we aim to understand the reason for this lack of attention, with particular emphasis on women of reproductive age, and to analyse the policy context in which homeless women’s health and well-being needs are situated.

## Methods

### Study setting

The fieldwork for this study was conducted in Addis Ababa, the capital city of Ethiopia, from August to September 2021. As the largest city in the country, it has a population of approximately 5 million people [54]. The population of Ethiopia was 117 million in 2021, placing it 2^nd^ in Africa and 12^th^ in the world [4]. By 2030, the population is projected to reach 122.3 million [54]. Thus, the number of homeless individuals will likely grow [55].

A study by the Addis Ababa City Administration’s Bureau of Labor and Social Affairs indicated that 92 per cent of homeless people in the capital come from other regions [56]. Most people who beg on the streets are estimated to come from the Southern Nations, Nationalities, and Peoples’ Region (SNNPR), the Oromia, and the Amhara Regional States.

Almost 51,000 street children and beggars are estimated to live in Addis Ababa, mostly between 13 and 45. However, the number is likely much higher. Only 8% of homeless individuals were born and raised in Addis Ababa [56]. There is no specific nationwide proportion of homeless women in Ethiopia. However, according to a study in one of the large cities of Ethiopia, the sex ratio is close to 50% [6].

### Study design and participants

The study employed a descriptive qualitative study design [57], which lets us comprehend a phenomenon, a process, or the perspectives and experiences of those involved. Standards for Reporting Qualitative Research and the consolidated criteria for reporting qualitative research were used [58, 59].

Thirty-four participants from governmental and non-governmental organisations participated in this study. The study included healthcare administrators and care providers, team leaders and staff from the Labour and Social Affairs Bureau, coordinators from the Service Users’ Association, a director from the Women and Children’s Affairs Bureau, social workers, program coordinators, and the initiators of charitable organisations, and health practitioners.

### Data collection technique and procedure

We selected the participants for this study using a purposive sampling method [60, 61], based on their knowledge, experience, profession, and position in service delivery and administrative functions related to homelessness in Addis Ababa. Their ability to provide detailed and in-depth information regarding the phenomenon was also considered.

We used snowball or chain sampling to select the final participants [61]. First, the fourth author (YB) requested permission to conduct this study in the selected organisations. Next, the first author (KY) and research assistants scheduled appointments with the organisation’s managers and department heads to give them an overview of the study. From each organisation, we selected departments closely linked to homeless services and individuals with experience in organising campaigns and providing services for homeless individuals. Participants were then scheduled for a discussion at a time that was convenient for them.

Following the completion of data collection from each organisation, the researchers asked the participants if they knew of other individuals or organisations that could be helpful. They were asked to suggest someone with experience in homeless-related services, campaigns, and programmes. After passing the researcher’s details on to the recommended person within the organisation, the researchers contacted the individual.

As part of the sampling process, we relied on our judgement to identify and select individuals we believed would provide the most helpful information for the study. Various organisations and participants with different disciplines and expertise were included to reduce response bias and to allow for a greater generalisation of the results. It took over two months to complete the comprehensive data collection and ethical process.

Moreover, our selection involved identifying and selecting individuals with specific knowledge and experience in homelessness care and support. However, the researchers’ judgements were carefully considered to maintain credibility, and the selection criteria were clearly outlined. Nevertheless, the selection of participants for the study might have been biased due to our professional and research experience, which is discussed under the ‘Reflexivity’ section.

In-depth interviews were conducted, lasting approximately one hour, with individuals at their workplaces. The interviews were performed in a private room where no one could observe or overhear the discussions. The first author and the research assistants conducted all the interviews in Amharic, the official national language of Ethiopia. The data were collected using an interview guide. The interviews were audio-recorded. The data collection was terminated when no additional issues or insights were identified.

### Data management and analysis

The research assistants were trained prior to the fieldwork. In addition, we triangulated the data sources, data collection methods and investigators to enhance the credibility of the research. For example, more than one investigator collected and analysed the data. The triangulation in the data collection method included combining in-depth interviews with secondary data. The initial data transcription was verbatim in Amharic and translated into English by the first author and the research assistants. The first and fifth authors (KY and SHvW) independently coded the interviews. The deductive thematic analysis was conducted using Shiffman and Smith’s framework with NVivo 12. We followed the Consolidated Criteria for Reporting Qualitative Research to ensure comprehensive reporting.

The first author, the field worker, has previous experience conducting research in the same context. The last author has extensive research expertise in qualitative research. These two authors discussed the framework thoroughly beforehand, and there were frequent debriefing sessions during the research. Our main aim was to present the participants’ perspectives accurately without introducing any biases due to our prior knowledge. For example, we spent over two months in the field, which included gaining ethical consent. A team of interdisciplinary co-authors with diverse research experience crosschecked the data and our interpretations. We presented our preliminary findings to other academics not involved in the research and collected feedback and insightful comments from them (Welfare Research Group, Department of Sociology, Uppsala University). The field researchers took notes and compiled memos during the research process.

### Ethical approval and consent to participate

The Institutional Review Board of the Addis Continental Institute of Public Health (ACIPH) approved this study. The registration number is ACIPH/IRB/009/2021. We obtained permission from the Addis Ababa Health Bureau to conduct interviews. Participants were informed of the purpose of the study. All participants signed an informed consent form prior to each interview. All interviews were conducted privately to protect participants’ confidentiality. We informed participants that the prolonged interview period would cause minimal discomfort. Reports and other documents did not contain the names of participants. The prevailing Coronavirus Disease 2019 (COVID-19) protocol was observed. The authors declare that the study was conducted following the Declaration of Helsinki.

## Findings

### Characteristics of the participants

In-depth interviews were conducted with 34 participants with diverse sociodemographic backgrounds. More than half of the participants were healthcare providers (*n* = 20).

More than half the participants had a master’s degree or had completed speciality medical training (*n* = 18) (Table 1).

**Table 1:** Descriptive characteristics of the participants (*n* = 34)

***Characteristics***		***Number of participants***
*Age*	20–29	6
	30–39	17
	40–49	8
	50–59	2
	60–69	1
*Gender*	Female	13
	Male	21
*Educational status*	Bachelor’s degree	14
	Master’s degree	15
	Medical specialty	3
	Diploma	2
*Profession*	Healthcare professional	14
	Psychologists	6
	Social worker	5
	Leadership and management	9
*Institution of the participant*	Government office	10
	Public hospital	14
	Mental Health Service Users Association	2
	Charity organization	8

Most participants described themselves as government employees (*n* = 24). The remainder (*n* = 10) reported that they worked for a non-governmental organisation or an association.

Table 2 summarises the major themes identified in the deductive thematic analysis according to the Shiffman and Smith framework.

**Table 2:** Factors shaping the political priorities to address the health and well-being of street homeless women in Addis Ababa, Ethiopia

Themes	Subthemes (Factors shaping political priority)
Theme 1: Actor power	1. Limited link-up of activity despite the presence of many actors 2. Absence of unifying leadership 3. Inefficiently organised and poorly coordinated tasks 4. Insufficient advocacy and mobilisation to pressurise national leaders into action
Theme 2: Ideas	1. Lack of consensus on how to address the needs of homeless women 2. Homeless women’s specific needs are not acknowledged or well-framed 3. Homelessness is mainly considered a security concern for the public
Theme 3: Political context	1. Homeless health is not explicitly addressed in national government policies and guidelines 2. Weak national governance structure
Theme 4: Issue characteristics	1. Lack of insight into the extent of the problem 2. Ineffective monitoring system 3. Lack of clearly defined and cost-effective interventions 4. Scarcity of resources

#### Theme 1 actor power

In the first theme, the actors involved in addressing the needs of homeless women are described (Table 3). This study identified a lack of unifying leadership for tackling homeless women’s health and well-being issues, which was critically needed to facilitate collaboration and cohesion among policymakers and the other actors. Additionally, no champion was available to advocate for homeless women, resulting in a lack of bottom-up pressure. The factors are described below.

**Table 3:** The type of organizations and actors that address homeless people in Addis Ababa, Ethiopia

Type of organization	Name of the organizations
**Government Offices**	The Addis Ababa City Administration Labor and Social Affairs office
	The Ministry of Women and Social Affairs
	Bureau of Transport
	The Addis Ababa City Administration health bureau
	The women and children affairs office
	Social rehabilitation enterprises
	Technical and vocational schools
	The justice system office including the police
	The Mayor's office
**Inter-governmental organization**	UNICEF The World Bank
**Public hospitals**	St. Amanuel Mental Specialized Hospital
	Eka Kotebe general hospital
	St. Paul Millennium medical college referral hospital
	Tirunesh Beijing hospital
	St. Petros hospital
	Zewditu Hospital
**Associations**	Addis Credit and Savings Associations
**Non-Profit** **Charitable Organization**	Born Again Mental Health Rehabilitation and Healing center
	The Macedonians Humanitarian Association (MHA)
	Gergesenon Mental Rehabilitation center
	Selihom charity center
	Mother Teresa Missionaries of Charity
	The Muday Association
	Rainbow Humanitarian Caretaker Foundation
	Hold My Hand
	Kibir Leargawuyan
	ZOA – Ethiopia- Meals, Emergency Shelters, Education
	AHOPE for Children
	Selam Children's Village
	Yezelalem Minch Children and Community Development
	Kidane Mehret Children's Home
	Sele Enat Mahiber
	Life Center Ethiopia
**International organizations**	UN World Food Programme
	World Vision Ethiopia
	Danish Refugee Council
	ActionAid Ethiopia
**Academia**	Addis Ababa University
**Volunteers**	Individuals, groups

##### Limited link-up of activity despite the presence of many actors

The list of participants indicated that a range of actors is involved in tackling homelessness issues and providing services to the homeless, including volunteers, public organisations, associations, international organisations, and charities (Table 3).

Several participants from the Labour and Social Affairs Bureau, public hospitals, and charity organisations reported that some of these actors had signed memorandums of understanding (MoUs) with public hospitals to deliver services or support. Furthermore, the Labour and Social Affairs Bureau team coordinator confirmed that they cooperate with charitable organisations.

*A memorandum of understanding was signed between the Labour and Social Affairs Bureau, healthcare facilities, and diverse organisations. In addition, the bureau has established partnerships with non-profit organisations (Interview #4, a government organisation).*

Despite this, most participants pointed out that there was limited and inconsistent collaboration, communication, and cohesion among the national network of individuals and organisations involved in tackling homelessness. In addition, the approaches taken by the different actors to address homelessness often differ (Interviews #2–4, 19–23, charity organisations and 29–31, government organisations).

##### Absence of unifying leadership

Participants from the various organisations emphasised that the stakeholders and individuals involved in homeless-inclusive interventions (see Table 3) seemingly lack a united leadership capable of bringing individuals and organisations together to tackle the phenomenon of homelessness and to address the needs of homeless individuals. Moreover, they pointed out that the city lacks collective leadership for addressing homelessness-related issues or homeless women’s health and social support. According to the participants, the absence of a robust unifying leadership makes it challenging to provide coordinated and organised services for homeless individuals (Interviews #1–4, #8–16, and #29–34, government organisations).

##### Inefficiently organised and poorly coordinated tasks

Participants from Addis Ababa City Administration’s Labour and Social Affairs Bureau explained that the government had assigned their office the responsibility of coordinating efforts to meet the needs of homeless individuals.

*As a government-mandated bureau [Labour and Social Affairs], our department [social rehabilitation] supports homeless individuals. Besides, these services are a part of the National Social Protection Policy, which includes guidelines and strategies for dealing with communities facing severe social crises (Interview #4, a government organisation).*

The services for homeless individuals involve removing people from the street through police roundups in the middle of the night and relocating homeless children and adults to centres outside the capital, where they can receive life-skills training and assistance, including food and shelter (Interviews #1–4 a government organisation).

However, some participants suggested that the current interventions are insufficient to meet the health needs of those with mental and psychosocial issues. Although the bureau has established several networks to address this issue, the services provided are limited to fulfilling homeless individuals’ basic needs, repatriation, life-skills training, referrals, rehabilitation, and reunifying institutionalised homeless individuals with their families. In addition, the participants discussed the difficulties of maintaining such a network over time (Interviews #1–4, a government organisation).

*Several services are provided to street individuals, including food, shelter, clothing, employment, life-skills training, and microfinance, as well as collaboration with local organisations to assist them in finding employment (Interview #2, government organisation).*

The Labour and Social Affairs Bureau staff stressed the importance of providing healthcare for the homeless. Indeed, the bureau provides periodic healthcare to homeless individuals alongside some public health institutions and charitable organisations (Interviews #1–4, government organisations).

However, no guiding organisation is specifically focused on meeting the health needs of homeless women and integrating those needs into their programmes, according to the participants (Interviews #1–4, government organisation).

##### Insufficient advocacy and mobilisation to pressurise national leaders into action

The actor category also encompasses civil society mobilisation. Participants expressed concerns about the lack of national advocacy for homeless communities.

Despite this, several humanitarian associations and Non-governmental organisations (NGOs) have participated in grassroots activities, including Meseret Humanitarian Organization (MHO), the World Bank, Mekedonia Humanitarian Association (MHA), and the United Nations Children’s Fund (UNICEF) (Table 3).

These organisations have been actively involved in addressing the needs of homeless people in different age groups and with varied health conditions. However, except for a few active volunteers on social media and charity organisations, there are no national-level advocates dedicated to the health and well-being of individuals living on the streets (Interviews #3, 4, 29, 33, government organisations, and Interview #11, a service users’ association). While undoubtedly true, some participants also expressed concern that civil societies and NGOs did not exert sufficient pressure on the political authorities to address the health and well-being of homeless women.

#### Theme 2 ideas

The participants involved in delivering homeless women’s health services disagreed about the solutions to the issues. Homeless women’s health and well-being needs have not yet been well-established and agreed upon. Additionally, homeless women are primarily stigmatised in society, and homeless individuals are often considered a safety concern. Some participants even commented that homeless individuals pose a security risk to the community due to their use of drugs and involvement in criminal activity.

##### Lack of consensus on how to address the needs of homeless women

The Labour and Social Affairs Bureau members emphasised the importance of addressing the basic needs of the homeless and rehabilitation measures over prevention, promotion, and early disease detection. Nevertheless, the actors involved in the issues around homelessness could not reach a consensus regarding the specific needs of homeless women and how they should be addressed.

*Our (Labour and Social Affairs Bureau) service delivery strategy begins with removing the homeless from the streets. Food, clothing, shelter, and medical care are all provided at the rehabilitation facility (Interview #4, a government organisation).*

On the other hand, some participants highlighted the urgent need to address the immediate health problems of homeless individuals (Interviews #8, 11, 29, 33, and 34).

In this regard, stakeholders and healthcare providers did not agree on which solution was the most appropriate for addressing the needs of street homeless women. Despite this, some participants emphasised the importance of rehabilitation (Interviews #7–10/#15, government organisations; interviews #29–34, public hospitals).

##### Homeless women’s specific needs are not acknowledged or well-framed

The participants discussed different perspectives regarding the health needs of homeless women. They focused on basic needs like food, clothing, and shelter. There was also an emphasis on the importance of healthcare (Interviews #1–4/#6–9/#15, government organisation; #29–34, public hospitals; #20–25, charities).

Some participants pointed out the serious issues around violence against homeless women, including attempted or completed rape, assault, and emotional or psychological violence (Interviews #4/8/13–17/29–32, government organisations; interviews #20–24, charities).

*Many homeless women have experienced violence, sexual abuse, and harassment and suffer psychological, physical, and emotional problems. It is challenging to give birth on the streets. Women who have been raped let their children beg as a source of income (Interview #14, a government organisation).*

Participants further indicated that the community tends to stereotype homeless women, resulting in them neglecting their needs. Although participants acknowledged the insecurities and challenges faced by homeless women on the street, some of them failed to highlight the specific needs of such women.

##### Homelessness is mainly considered a security concern for the public

Some participants in this study described the homeless as a security problem rather than a marginalised population (Interviews 4/#8–10/#11/#15, government organisation; #29–34, public hospitals).

*Homeless individuals often do not wish to return home; some do not have a family but feel uncomfortable on the streets. Substance addiction may be a problem for some of these individuals. Additionally, it may threaten the future peace and security of the communities in which they live (Interview #8, government organisation).*

#### Theme 3 political contexts

Participants discussed the political and organisational environments in Addis Ababa. The following subthemes were discussed concerning the political context.

##### Homeless health is not explicitly addressed in national government policies and guidelines

There was an inadequacy in meeting the health needs of homeless individuals, as explained in the background section. Moreover, there is a lack of a clear system for supporting the health needs of homeless women. There also seems to be a lack of enthusiasm for this topic and only limited political support, except for a few Ethiopian politicians and hospital administrators, who are recognised for their campaigns to address homelessness. Even though the efforts of these latter mentioned actors, their campaigns were not sustainable, according to the participants.

##### Weak national governance structure

Participants discussed the existence of international and national strategies, policies, and health-specific strategies. One of the programmes highlighted was the mental health gap (mh-GAP) training. This has been offered to non-specialised health professionals since 2010 as part of WHO’s strategy to integrate mental health services into primary care. However, these services do not include mental health services for the homeless.

*Services were made available to those who could visit the hospital. There is also a National Mental Health Strategy, although the homeless are not considered (Interview #15, a government organisation).*

Participants stressed the lack of structural support and sustained action platforms for homeless individuals in the healthcare sector. A mental health service users’ association (MHSUA) is also present in Addis Ababa; however, this association does not address the needs of the homeless. Further, little evidence was found that health extension workers target the homeless.

*As of now, we have not begun to address homelessness. We want to help others. Our association recruited a recovering mental health patient who understands our mission and can relate to our beliefs. (Interview #11, MH service users’ association).*

Despite concerns about resource scarcity and the lack of governance structure, the primary care unit team leader of the Addis Ababa health bureau mentioned initiating outreach services in the city.

*As part of the health bureau’s Urban Health Extension and Primary Health Care programme, eight to twelve doctors provide home-based care. In contrast, others provide outreach services to the homeless [Interview #16, government organisation].*

#### Theme 4 issue characteristics

According to some study participants, three aspects of the issue hindered efforts to garner support for addressing the health and well-being of homeless women. There are no indicators, no documentation of the service gaps and the magnitude of the problem, and the current response has been ineffective. Additionally, no well-established intervention has been presented to address homeless women’s health and well-being needs.

##### Lack of insight into the extent of the problem

Participants agreed that homelessness issues persist despite the ongoing support available and the efforts of many actors. In addition, the study participants expressed concern about the lack of data on the size of the burden concerning the health conditions of homeless women and their unmet health needs (Interviews #1–4–6/#12/#15–17, government organisation).

*Because of the lack of sufficient data on the number of homeless women, the research findings, and the extent of the problem, the health needs of homeless women have been neglected in the policy agenda. Therefore, the extent of the problem is poorly understood (Interview #16, a government organisation).*

##### Ineffective monitoring system

A few Labour and Social Affairs Bureau participants expressed concerns that the monitoring system for the ongoing interventions for homeless individuals was ineffective (Interviews #2–4, government organisation).

Participants emphasised that measures to monitor the progress and severity of the problem and insights from previous activities on monitoring the effectiveness of interventions were not clearly understood or shared. Two individuals with supervisory and team leader experience in mental health services and social rehabilitation also expressed concerns regarding the lack of data and indicators regarding mental health among the homeless and coordinated programme evaluation (Interviews #3 and #16, government organisation).

*Rehabilitation has not generally been effective or successful for me. Several institutionalised individuals failed rehabilitation programmes and were forced to move onto the streets. A lack of interest has been expressed in collaborative projects, no joint assessments have been conducted, and neither government nor non-governmental organisations have conducted monitoring. Rehabilitation has not progressed as rapidly as initially anticipated (Interview #2, government organisation).*

Moreover, the Labour and Social Affairs Bureau participants indicated that the lack of a sustainable evaluation system prevented them from adequately evaluating and following up on the services provided to homeless individuals (Interviews #1#2#3, government organisation).

##### Lack of clearly defined and cost-effective interventions

Strategies for addressing the issues of homeless women were not clearly explained. Considerable emphasis was placed on the complexity and multidimensional nature of the issues related to the health of homeless women. In addition, participants discussed the complexity of implementing campaigns and ongoing interventions, indicating that the participants were uncertain about the most effective approach.

Several participants reported that charitable organisations collaborated with government hospitals to provide healthcare services. These involved primarily curative or rehabilitation services, though. Among the participants, some preferred rehabilitation to preventive and curative care. However, some participants explained that there were no clearly explained, cost-effective, and inventive approaches to address the issue of homelessness in light of the limited resources available (Interviews #4/9/11–16/29–32, government organisation; #20–23, charities).

In addition, several participants acknowledged that the interventions needed to make a difference were complex and that the community’s insurance coverage for homeless women was unavailable. Participants from the Labour and Social Affairs Bureau emphasised this point as follows:

*Homeless people lack sufficient financial resources to cover these expenses (rehabilitation costs), and community health insurance does not cover these expenses unless the individual is institutionalised (Interview #4, government organisation).*

##### ***Scarcity of resources***

Several participants mentioned that most patients have to pay for their health care out of their own pocket. Moreover, government organisation’s services were not inclusive, free access was not available, and the needs of women in different government sectors were generally not taken seriously.

*Organisers of the public hospital have requested financial assistance from the Minister of Health to provide mental health services to the homeless. Despite this, they did not fund the programme, which was one of the factors that compromised the sustainability of the campaign (Interview #29, government organisation).*

## Discussion

Our study investigates why policies and ongoing programmes rarely address homeless women’s health and well-being needs. For this purpose, we applied the four categories of factors described in the framework to assess the political priority determinants [30]. The policy community in Ethiopia lacks unity, bottom-up pressure, and sustainable, co-ordinated tasks regarding actor power. Therefore, the different organisations are not able to obtain political priority for the issue of the health and well-being of homeless individuals.

Despite their best efforts, there is limited co-ordination among homeless women’s health and well-being support providers. A study by Shiffman indicates that a lack of co-ordination and leadership can weaken the actors’ ability to gain political support for a programme. This finding is consistent with a study conducted in Kenya [62].

According to this study, a lack of entrepreneurs to provide strong leadership negatively shaped the political prioritisation of adolescent sexual and reproductive health [62]. The findings of this study are consistent with those of studies carried out in five nations: Ghana, Guatemala, India, the Philippines, and Salvador [63]. According to the study, stakeholders in Ghana lack cohesion [63].

Further, the findings of this study are consistent with research on the generation of political priorities for mental health and global surgery [64, 65].

Service providers and programme leaders tend to conceptualised homeless women’s health and well-being needs and the solutions differently. In particular, the lack of consensus regarding the priority areas and intervention types has made it difficult for stakeholders to unite and attract political attention. Creating consensus about how problems and their solutions are presented internally and externally is crucial to gaining political support and governing effectively [30]. Results from generating political priorities for global surgery and mental health agree with our findings [64, 65].

Homelessness has increased recently, including among women [66]. Several factors contribute to the vulnerability of homeless women [67, 68]. In addition to unplanned pregnancies [69], homeless women experience unsafe abortions [70], loss of reproductive health rights [71, 72], and reproductive and sexual health-related traumas [71].

Despite the evidence of these elevated risks, some of the participants in our study failed to focus on homeless women’s health-care needs, let alone their specific maternity care needs.

In addition, most national health and social protection documents fail to address the specific needs of homeless women. Although the family planning guideline [45] mentions that homeless women need innovative family planning approaches, no other documents, directions, or strategies mention homeless people. Rehabilitation is also emphasised in the mental health strategy [48]. However, our document review shows that the existing health and social protection policies and guidelines do not explicitly mention the health and well-being of homeless women. This issue needs to be addressed and given political priority.

Concerning the policy window, the United Nations Sustainable Development Goals (SDGs) recognise the importance of women’s health, gender equity, well-being, and reproductive rights [73]. Moreover, SDG 5 aims to achieve gender quality and empower all women and girls. Specifically, SDG 5.1 aims to end all forms of discrimination against women and girls everywhere [73], while the focus of SDG 5.6 is on ensuring universal access to sexual and reproductive health and reproductive rights. Besides this, SDG 3 focuses on ensuring healthy lives and promoting well-being for all ages. This is relevant because, in addition to living in severe conditions, homeless women are at increased risk of maternal morbidity and mortality [14].

However, none of the 17 SDGs or their 169 indicators mentions homelessness [74]. This shows how limited is the political attention given to the health and well-being of homeless individuals, and will negatively affect the ability to meet SDGs 3 and 5.

Although homeless women are at high risk of suffering from poverty, hunger, poor health, and a lack of access to education, clean water, and sanitation [72, 75], they are overlooked not only in SDGs 3 and 5 but also in SDGs 1.4, 1.5, 8.5, 10 (Reduce Inequalities), and 11 [73]. Furthermore, homelessness also contributes to rising inequality and hinders the development of sustainable and inclusive cities [76]. In this manner, nearly all of the SDGs might be undermined if homelessness as an issue is not addressed. Hence, an initiative to include homelessness in the SDGs was initiated nationally and internationally [68]. One example is the United Nation’s Working Group on Ending Homelessness (UN-WGEH) [77]. WGEH includes about 30 organizations, including the Institute of Global Homelessness [74].

Several challenges confront people experiencing homelessness, making it difficult to find straightforward, scientifically proven, and implementable innovative solutions. Several participants in this study highlighted the difficulty of finding effective interventions. A similar finding was reported in another study from Kenya [62].

This study indicates that homelessness is a complex issue that is difficult to quantify and associated with several reproductive and social outcomes for women. It aligns with the results of a previous study identifying the challenges associated with addressing elder abuse in poor urban settings [78]. Further, there are no public health statistics regarding the extent and severity of the problem, making it difficult to quantify and represent. As a result, the issue has been relegated to a lower priority on the political agenda.

Policymakers, funders, and politicians may have difficulty recognising the need for a multi-sector response without a comprehensive understanding of the scope of the problem [30, 40]. According to two studies on global health and HIV/AIDS, gaining political priority requires public positioning that is aligned with public opinions, evidence, and current programmes [79, 80]. With this in mind, it has been suggested that a way to gain political support is to consider the recommendations of policymakers, non-governmental organisations, and other external funding agencies. However, although various actors might be involved [81], their responsibilities are not equitably distributed. Additionally, there is no consensus about who should address the health and well-being of homeless people. While this is the case, the Labor and Social Affairs Bureau has several ongoing activities that may support effective governance. There are other reasons to be optimistic, too. In conjunction with the newly formed family teams, an urban health extension programme is addressing some of the health concerns of homeless individuals.

### Strengths and limitations of the study

A well-established framework guides the analysis. However, the framework developed by Shiffman and Smith does not address the non-implementation of legislation after it has been passed. Instead, it proposes practical steps for improving the situation. We included participants from governmental and non-governmental organisations and service users’ associations for this study. This allowed us to gain a holistic perspective on the situation. However, this study focused exclusively on the perspectives of programme managers, charity organisation initiators, leaders, and care providers; it did not involve country-level policymakers, programmers, or politicians, which could be a limitation.

## Conclusions and recommendations

As Shiffman and Smith noted previously, an issue is high on government agendas when the following conditions are met: national actors actively participate in a topic, policies are enacted using authoritative decision-making processes, and resources are available [30]. This study indicates that homeless women’s health and well-being needs are not being adequately addressed in Addis Ababa, Ethiopia. A lack of cohesion and unifying leadership within the policy community is hindering the political prioritisation process. In addition, the absence of clear governance structures and an inability to quantify the health burden of this population have made it difficult for issues to be prioritised. Consensus has not been reached regarding how this issue should be framed and presented. In Ethiopia, health policy documents and strategies do not adequately address issues relating to the health and well-being of homeless women. Future strategies, studies, and programmes must be directed at assessing and quantifying the health and well-being of Ethiopia’s homeless women. Raising the political priority of homeless women’s health in the policy arena would allow more effective approaches to be developed. It is also essential to have integrated social protection programmes to alleviate health insecurity, poverty, and vulnerability in the health-care system. Moreover, developing concepts and interventions based on the political system and providing gendered services would be beneficial in addressing the health and well-being of homeless women. In addition, all stakeholders should establish more robust networks of champions and frameworks to ensure support for the homeless, including homeless women, across all sectors. Such a network would provide an opportunity to develop innovative, comprehensive, and scientifically proven approaches to addressing homeless women’s health and well-being needs.

## Reflexivity

Two of the authors of our study, KY and YB, are Ethiopian citizens and thus especially familiar with the study’s context. KY worked previously as a mental health practitioner in Ethiopia. Furthermore, five of the research assistants were health professionals who work at some of the governmental and non-governmental organisations included in the study. Without this insider knowledge, we may not have recognised the need to involve participants beyond health-care and programme co-ordination.

The previous work experience of both KY and the data collectors may have influenced the data design, collection, and analysis. To address this, we kept careful logs of the research process. We set regular meetings at which we reflected on our views and experiences and how they may influence the study.

In addition, we enhanced the study’s credibility by involving researchers from various disciplines to offer a broad conceptualisation of homeless women’s health and well-being, besides having extensive experience in qualitative research in different study contexts. Furthermore, all steps involved in collecting, analysing, and interpreting the data were carefully considered. Moreover, we described thoroughly how the research procedure was undertaken to provide a comprehensive description. In the discussion, we compared our findings with existing research studies, concepts, and practices.

## Data Availability

The data underlying this article cannot be made publicly available due to respect for the privacy of the individuals who participated in the study and their roles within the organisations they represent. It is possible to obtain the datasets used and analyzed during the current study from the corresponding author upon reasonable request.

## Abbreviations

ACIPH: Addis Continental Institute of Public Health
MHA: Macedonians Humanitarian Association
mh-GAP: Mental Health Gap
SDGs: Sustainable Development Goals
STDs: Sexual Transmitted Diseases
UNICEF: United Nations Children’s Fund
WGEH: Working Group on Ending Homelessness
